# Soluble LDL-receptor is induced by TNF-α and inhibits hepatocytic clearance of LDL-cholesterol

**DOI:** 10.1007/s00109-023-02379-4

**Published:** 2023-10-20

**Authors:** Mulugeta M. Zegeye, Sravya S. Nakka, Jonas S. O. Andersson, Stefan Söderberg, Liza U. Ljungberg, Ashok K. Kumawat, Allan Sirsjö

**Affiliations:** 1 Cardiovascular Research Centre, School of Medical Sciences, Örebro University Södra Grev, Rosengatan 32, 703 62 Örebro, Sweden; 2 Department of Infectious Diseases, Sahlgrenska University Hospital, Region Västra Götaland, Gothenburg, Sweden; 3 Department of Public Health and Clinical Medicine, Skellefteå Research Unit, Umeå University, 931 86 Skellefteå, Sweden; 4 Department of Public Health and Clinical Medicine, Medicine Unit, Umeå University, Umeå, Sweden

**Keywords:** Hypercholesterolemia, Chronic inflammation, Myocardial infarction, Mediation analyses, ADAM-17, MMP-14

## Abstract

**Abstract:**

Defective LDL-C clearance and hence its elevation in the circulation is an established risk factor for cardiovascular diseases (CVDs) such as myocardial infarction (MI). A soluble LDL-receptor (sLDL-R) has been detected in human plasma which correlates strongly with circulating LDL-C and classical conditions that promote chronic inflammation. However, the mechanistic interplay between sLDL-R, inflammation, and CVDs remains to be investigated. Here, we report that stimulation of HepG2 cells with TNF-α induces the release of sLDL-R into culture supernatants. In addition, TNF-α induces gene expression of peptidases ADAM-17 and MMP-14 in HepG2 cells, and inhibiting these peptidases using TMI 1 significantly reduces the TNF-α induced sLDL-R release. We found that a soluble form of recombinant LDL-R (100 nM) can strongly bind to LDL-C and form a stable complex (KD = E-12). Moreover, incubation of HepG2 cells with this recombinant LDL-R resulted in reduced LDL-C uptake in a dose-dependent manner. In a nested case-control study, we found that baseline sLDL-R in plasma is positively correlated with plasma total cholesterol level. Furthermore, a twofold increase in plasma sLDL-R was associated with a 55% increase in the risk of future MI [AOR = 1.55 (95% CI = 1.10–2.18)]. Nevertheless, mediation analyses revealed that a significant proportion of the association is mediated by elevation in plasma cholesterol level (indirect effect *β* = 0.21 (95% CI = 0.07–0.38). Collectively, our study shows that sLDL-R is induced by a pro-inflammatory cytokine TNF-α via membrane shedding. Furthermore, an increase in sLDL-R could inhibit hepatic clearance of LDL-C increasing its half-life in the circulation and contributing to the pathogenesis of MI.

**Key messages:**

- TNF-α causes shedding of hepatocytic LDL-R through induction of ADAM-17 and MMP-14.
- sLDL-R binds strongly to LDL-C and inhibits its uptake by hepatocytic cells.
- Plasma sLDL-R is positively correlated with TNF-α and cholesterol.
- Plasma sLDL-R is an independent predictor of myocardial infarction (MI).
- Plasma cholesterol mediates the association between sLDL-R and MI.

**Supplementary Information:**

The online version contains supplementary material available at 10.1007/s00109-023-02379-4.

## Background

Elevated low-density-lipoprotein cholesterol (LDL-C) in the circulation is an established risk factors for cardiovascular diseases (CVDs) [1] which are the leading causes of death, claiming the lives of 17.9 million people worldwide (World Health Organization (WHO) 2019 report). LDL-C accumulates in blood vessel walls causing inflammatory responses and gradual thickening and hardening of arteries, a process known as atherogenesis [2]. One of the major causes of elevated LDL-C in blood is a defective clearance by the liver, which under normal circumstances, is achieved through LDL-receptor (LDL-R) mediated endocytosis of LDL-C [3].

LDL-R is a single-pass trans-membrane receptor that is abundantly expressed in several cell types making up the liver, adipose tissue, and endocrine organs [3]. It is composed of 839 amino acids arranged in five modules including the LDL receptor type A repeats, the EGF precursor (EGFP) homology domain, and trans-membrane segment and cytosolic tail [4]. The LDL receptor type A repeats is the ligand biding domain which is made up of the first 292 amino acids on the N-terminus [5]. Upon binding of LDL-C, the receptor-LDL-C complex gets internalized via clathrin-mediated endocytosis [6]. The low pH in the endosome allows the release of LDL-C from LDL-R after which the LDL-R gets recycled back to the plasma membrane while the LDL-C continues its journey in the maturing endosomal system [7, 8]. As such, the LDL-R makes several hundreds of trips into and out of the cell roughly in every 10 min throughout its 20-h lifespan [7].

Expression of LDL-R is tightly regulated by transcriptional and post-transcriptional factors that are in turn regulated by availability of intracellular cholesterol. Gene transcription of LDL-R is mainly driven by the transcription factor SREBP2 (sterol regulatory element-binding protein 2) which is activated by low availability of intracellular cholesterol [9]. Activated SREBP2 translocate into the nucleus and upregulate genes that increase de novo synthesis and exogenous uptake of cholesterol [10, 11]. Whereas, during excessive abundance of intracellular cholesterol, SREBP2 is inactivated, and the LDL-R level is down-regulated via post-transcriptional actors such as PCSK9 (proprotein convertase subtilisin/kexin type 9) and IDOL (inducible degrader of LDL-R) that promote lysosomal degradation of the receptor [12, 13].

There is a soluble form of the LDL-R (sLDL-R) in the blood, and prior studies have shown that it is strongly correlated to circulating lipid profiles [14–16]. In addition, the sLDL-R in blood has been associated with classical factors that promote chronic inflammation such as age, sex, and obesity [17]. These, in addition to the well-studied role of pro-inflammatory cytokines in lipid metabolism, have given rise to speculations that inflammation might have a direct role in the release of sLDL-R. Moreover, the mechanism behind the positive correlation between sLDL-R and LDL-C in the circulation, and its impact on cardiovascular diseases remains elusive.

In the current study, we report that the pro-inflammatory mediator TNF-α causes shedding of membrane-bound LDL-R from human hepatoma (HepG2) cells through increased activity of peptidases ADAM-17 and MMP-14. In addition, we demonstrate that a soluble form of LDL-R can significantly reduce LDL-C endocytosis by human hepatocytic cells. Furthermore, using a nested case-control study, we provide evidence that plasma level of sLDL-R at baseline was strongly associated with future myocardial infarction (MI) and that a significant proportion of the association can be explained by elevation in plasma cholesterol level.

## Methods and materials

### Cell culture

HepG2 cells (Merck KGaA, Germany) were cultured in 75-cm^2^ flasks (Sarstedt, Germany) containing Advance-MEM medium (AMEM, Gibco, Life Technologies, USA) supplemented with 10% FBS (Gibco, Life Technologies, USA) and 2 mM L-glutamine (Gibco, Life Technologies, USA). Antibiotics (penicillin (0.1 U/ml) + streptomycin (100 ng/ml)-PEST, Gibco, Life Technologies, USA) was included in the medium during culturing of HepG2 cells except during stimulations. The cells were kept at 37 °C and 5% CO_2_ environment and the medium were replaced every 48–72 h.

### Treatments of HepG2 cells

HepG2 cells were seeded in 24-well or 6-well plates at cell densities of 2 × 10^5^ or 1 × 10^6^ cells/well, respectively, in supplemented AMEM medium containing PEST. Following an overnight incubation, the medium was replaced with fresh antibiotics-free medium, and cells were stimulated with recombinant human TNF-α (R&D systems, USA) for 30 min to 48 h. In addition, inhibitors UK383367 and TMI 1 (both from R&D systems, USA) were added with or without TNF-α to cells for 48 h. Culture supernatants and cells were collected at the end of incubation and used for downstream applications or kept at − 80 °C until further analysis.

### ELISA

The release of sLDL-R into culture supernatants was quantified using sandwich ELISA kit (DuoSet^®^, R&D systems, USA) following the manufacturer’s instructions. At the end of the assay, optical densities were recorded at 450 nm using Cytation 3 Imaging reader (BioTek, Winooski, USA).

### Flow cytometry

HepG2 cells were detached using EDTA and gentle scrapping. Next, cells were washed with PBS containing 1 mM EDTA and 2% FBS and stained with anti-LDL-R-APC (Abcam, UK) antibody for 25 min at 4 °C in the dark. 7AAD staining was performed to remove dead cells from analyses, and cells only stained with 7AAD were used as fluorescence minus one (FMO) control. Using Gallios™ Flow Cytometer (Beckman Coulter Life Sciences, UK), stained cells were acquired and analyzed using Kaluza flow cytometry analysis software version 1.3 (Beckman Coulter, UK).

### LDL-C uptake assay

HepG2 cells cultured in 24-well plates were rinsed with FluoroBrite™ DMEM (Gibco, Life Technologies, USA). Next, diluted pHrodo™ Red LDL in FluoroBrite™ DMEM at a concentration of 5 µg/ml with or without recombinant human LDL-R (rhLDL-R, R&D systems, USA) was added on to the cells. The cells were incubated for 3 h at 37 °C and 5% CO_2_ environment. At the end of incubation, the cells were analyzed with flow cytometry and fluorescence microscopy for endocytosis of LDL. Using Cytation 3 Imaging reader, bright field images and fluorescent signals produced with similar intensity of laser power were captured. For flow cytometry analyses, cells were detached using EDTA and gentle scrapping and were washed with PBS containing 1 mM EDTA and 2% FBS. FVS-BV510 (BD biosciences, USA) staining was performed to remove dead cells from analyses; and cells only stained with FVS-BV510 were used as fluorescence minus one (FMO) control. Stained cells were acquired using Gallios™ Flow Cytometer and analyzed using Kaluza flow cytometry analysis software version 1.3.

### RNA extraction/cDNA synthesis and qPCR

RNA was extracted from frozen cells using E.Z.N.A^®^ Total RNA Kit (OMEGA Bio-tek Inc, USA) following manufacturer’s instruction. The quality and quantity of extracted RNA was determined using NanoDrop™ 2000 (Thermo Fisher Scientific, USA) spectrophotometer. Synthesis of cDNA was achieved using 1 µg of total RNA and high-capacity cDNA reverse transcription kit (Thermo Fisher Scientific, USA). The reaction mix was prepared according to manufacturer’s instructions and allowed to run in the following thermal conditions: 10 min at 25 °C, 120 min at 37 °C, 5 min at 85 °C and kept at 4 °C before storage at − 20 °C. Gene expression analyses were performed using TaqMan™ qPCR primers/probes (Thermo Fisher Scientific, USA). The reaction mix contained TaqMan™ Fast Advanced Master Mix (Thermo Fisher Scientific, USA), TaqMan Primer/Probe water and cDNA in a 10-µl total volume. Using QuantStudio 7 Flex Real-time PCR system (Applied Biosystems, Foster City, USA), the reaction mix was run at 95 °C for 1 s and at 60 °C for 20 s for 40 cycles in addition to one step initialization at 95 °C for 20 s. GAPDH was used as housekeeping gene to normalize relative quantities recorded for each well.

### Biolayer interferometry analysis (BLI)-based affinity assay

Concentration dependent-binding kinetics of LDL-R to cholesterol LDL-C was analyzed using Biolayer Interferometry analysis (BLI)-based affinity assay. Briefly, 5 µg/ml of LDL-C (Life technologies, USA) diluted in acetate pH 4.5 (Cytiva, Sweden) was immobilized on amine coupling sensors (AR2G sensors; Sartorius AG, Germany). For immobilization step, sensor surface was activated by freshly prepared NHS-EDC mixture (Thermo fisher scientific, USA) and upon immobilization of LDL-C, ethanolamine (Cytiva, Sweden) was used to deactivate any unused surface of AR2G sensors. Different concentrations of rhLDL-R (0.01, 0.1, 1, 10, 30, 100, 1000 nM) diluted in PBS (Gibco, Life technologies, Sweden) were prepared and run as analyte in Octet K2 two channel system (Sartorius, Germany). Glycine (Cytiva, Sweden) at 10 mM concentration was used as regeneration buffer to regenerate sensors in between the sample runs. A typical run was set to 60 s of baseline using PBS, 300 s of immobilization (100 s of NHS-EDC and 100 s of Ethanolamine), 420 s of association, 420 s of dissociation, and 60 s of regeneration for each sample using the octet data acquisition software version 11.0 (Sartorius, Germany). On-rates and off-rates of rhLDL-R to immobilized LDL-C were measured, analyzed, and processed using 1:1 global curve fitting model using the octet data analysis software version 11.0 (Sartorius, Germany). KD was calculated from the ratio of kd to Ka and affinity of LDL-C is inversely proportional to KD (i.e., high affinity molecules have lower KD values). Sensograms of real-time binding kinetics were processed using trace drawer software version 1.9.2 (Cytiva, Sweden).

## Clinical study

### Study population and baseline clinical parameters collected

**Fig. 1:**
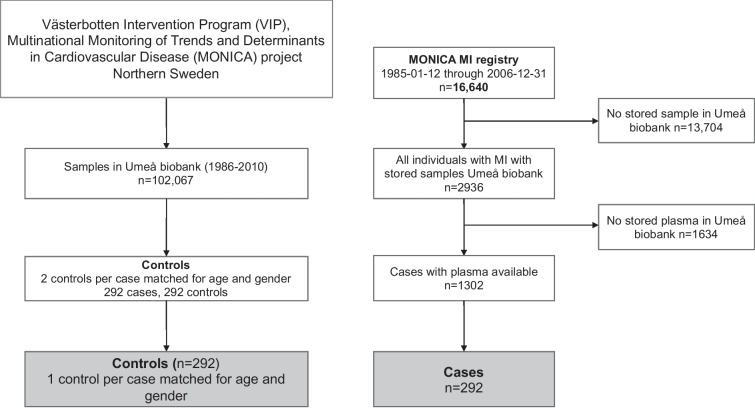
Flowchart showing the inclusion of study participants. The time from screening to incident MI in MONICA cohort ranged from 0.01 to 20 years with median of 6.9 years. The cases included in the current study were chosen depending on shorter duration between screening and incident MI (≤ 7 years), and one of the two matched controls of each case was randomly selected

A nested case-control was conducted by including 292 individuals with MI and 292 age- and sex-matched control individuals from the Västerbotten Intervention Programme (VIP) and the MONICA (Multinational Monitoring of Trends and Determinants in Cardiovascular Disease) study in northern Sweden (Fig. 1). The designs of the VIP and MONICA study are previously described in detail [18, 19]. Briefly, population-based risk factor surveys were conducted using a questionnaire in which participants were asked to provide information about their health-related lifestyle and cardiovascular risk factors. Anthropometry and blood pressure (BP) measurements were also recorded. In addition, venous blood samples were collected using heparinized glass tubes. Plasma samples prepared by centrifugation (1500 g for 15 min) were analyzed or stored at − 80 °C until further analyses. The plasma concentration of total cholesterol and blood glucose were determined using standard laboratory techniques. The study protocol was approved by the Regional Ethical Review Board in Umeå and complies with the Declaration of Helsinki.

In between the years 1985 and 2006, a total of 16,640 validated MI events were recorded based on symptoms, biomarkers, and electrocardiogram (ECG) recordings [19]. The individuals with MI were cross-checked whether they had participated in the population-based risk factor surveys. In addition, two control individuals with no previous MI were matched to every case based on sex and age (± 2 years). Date of blood sampling for controls was between ± 4 months that of cases.

### Operational definitions

The variables physical inactivity status, obesity, hypertension status, diabetes, and smoking status were dichotomized in the current study using the following definitions. Study participants who reported to never or occasionally exercise were categorized as “physically inactive,” while those who exercised at least once per week were grouped under “physically active.” Participants with body mass index (BMI) of ≥ 30 were grouped as “obese.” Participants with systolic BP ≥ 140 mmHg and/or diastolic BP ≥ 90 mmHg and/or taking antihypertensive medication were grouped as “hypertensive.” Diabetic participants were identified based on self-reported diabetes diagnosis or a fasting plasma glucose ≥ 7 mmol/L and/or 2-h post-prandial glucose in capillary blood ≥ 12.2 mmol/L (plasma glucose ≥ 11.0 mmol/L in MONICA). Based on their smoking habits at baseline, participants were categorized as “current smoker” and “non-smoker.”

### Measurement of sLDL-R and TNF-α

The plasma levels of sLDL-R and TNF-α were analyzed using Olink proteomics (Uppsala, Sweden) Cardiovascular III and Inflammation panels, respectively. The method is based on proximity extension assay (PEA) where a protein is targeted by two antibodies tagged with complementary nucleotide chains that can hybridize in the presence of the target protein and get amplified with PCR. The PCR product is proportional to target protein concentration in the sample, and the results are reported as normalized protein expression (NPX) on a log2 scale.

### Data analyses

In vitro data were analyzed using GraphPad Prism^®^ statistical software version 9.0 (GraphPad Software, USA). Data are presented as mean ± standard error of the mean (SEM) of at least 3 sets of experiments. For comparison between groups, paired *t*-test/Wilcoxon matched paired test, one sample *t*- and Wilcoxon tests, and one-way ANOVA for repeated measures followed by Bonferroni post-hoc test was used. *p*-value less than 0.05 was considered as statistically significant.

Data from the nested case-control study was analyzed using SPSS version 28 (IBM, USA). The distribution of categorical and numerical variables among cases and controls was compared using the chi-square test or Mann-Whitney *U* test, respectively. Similarly, comparison of plasma level of LDL-R among categories of baseline clinical characteristics was performed using Mann–Whitney *U* tests. Pearson’s correlation was performed between the plasma levels of LDL-R and total cholesterol as well as TNF-α. Association of plasma sLDL-R and other independent variables to MI was determined using uni-variable and multi-variable logistic regression analyses. Furthermore, mediation analysis was performed using the approach by Hayes and Preacher [20] to determine the direct and indirect effects of the association between plasma sLDL-R incidence of MI. In this analysis, total cholesterol level was used as a mediator. The proportions mediated were estimated using the approach for binary outcomes and logistic regression by Vandeweele TJ [21]. The mediation analyses were performed using Process version 3.5 added on to SPSS.

## Results

### TNF-α induces release of soluble LDL-R (sLDL-R) from HepG2 cells

**Fig. 2:**
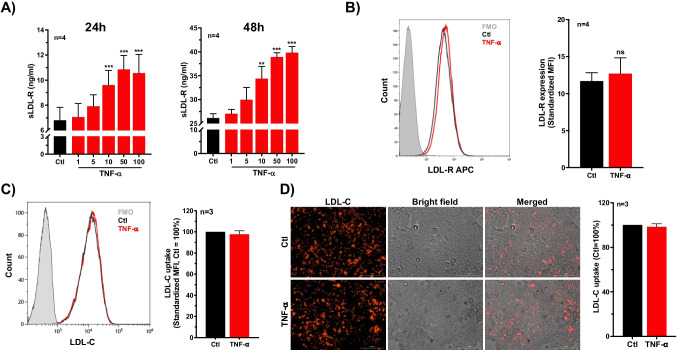
TNF-α induces release of soluble LDL-R (sLDL-R) in HepG2 cells. **A** Bar graphs showing a dose-dependent release of sLDL-R from HepG2 cells treated with TNF-α (24 h and 48 h) into culture supernatants. **B** A representative histogram and a bar graph showing the surface expression of LDL-R on HepG2 cells following TNF-α treatment (50 ng/ml for 48 h). **C** A representative histogram and a bar graph depicting the LDL-C uptake by HepG2 cells following TNF-α treatment (50 ng/ml for 48 h). **D** Representative microscopic images and quantification of LDL-C uptake by HepG2 cells treated or not with TNF-α (50 ng/ml) for 48 h. **p < 0.01, ***p < 0.001. AU arbitrary unit, FMO fluorescence minus one, MFI median fluorescence intensity, ns non-significant

To investigate whether TNF-α is involved in generation of sLDL-R, HepG2 cell were incubated with increasing concentration of recombinant human TNF-α protein. We found that stimulation of HepG2 cells with TNF-α induced significantly higher levels of sLDL-R release in culture supernatants compared to untreated controls. This response was dose-dependent and apparent at 24 h and 48 h of incubation (Fig. 2A). Similarly, TNF-α induces release of sLDL-R release from vascular endothelial cells, THP-1 cells, as well as PMA-differentiated THP-1 cells (Supplementary Fig. 1). Meanwhile, flow cytometry analyses of surface expression of LDL-R on HepG2 cells showed no significant difference in TNF-α stimulated cells (48 h) compared to unstimulated cells (Fig. 2B). Furthermore, we assessed the LDL-C uptake capacity of HepG2 cells using pHrodo Red labeled LDL-C that gives fluorescence signal only in endo-lysosomal environment. Briefly, following 48 h of incubation with or without TNF-α, HepG2 cells were rinsed and incubated with pHrodo Red labeled LDL-C for 3 h and analyzed for fluorescence signal. As presented in Fig. 2C, D, flow cytometric and microscopic analyses revealed that TNF-α treated and untreated cells showed comparable level of LDL-C uptake. Collectively, these findings indicate that TNF-α induces the release of sLDL-R from HepG2 cells with limited effect on membrane-bound LDL-R expression as well as functional consequence in terms of LDL-C uptake.

**Fig. 3:**
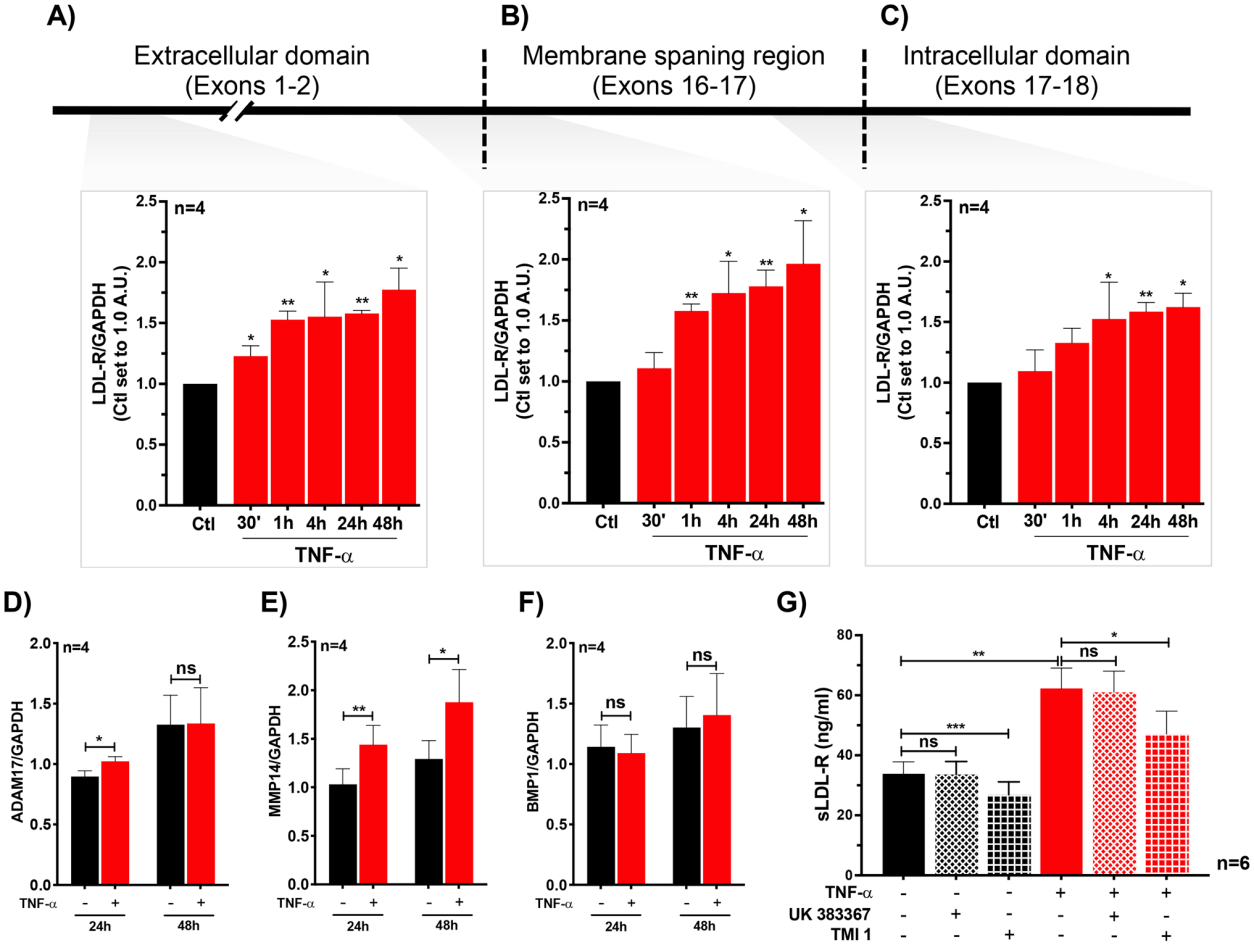
TNF-α induces the gene expression of LDL-R and surface protein peptidases in HepG2 cells. Bar graphs depicting gene expression of LDL-R variants in HepG2 cells treated with TNF-α (50 ng/ml) using primers targeting exons 1–2 (**A**), 15–16 (**B**), and 17–18 (**C**). Bar graphs showing gene expression of peptidases ADAM-17 (**D**), MMP14 (**E**), and BMP1 (**F**) in HepG2 cells treated with TNF-α (50 ng/ml). **G** Bar graph showing the level of sLDL-R released from HepG2 cells stimulated with TNF-α (50 ng/ml) in the absence or presence of peptidase inhibitors UK383367 (1 µM) and TMI 1 (1 µM). *p < 0.05, **p < 0.01, ***p < 0.001. AU arbitrary unit, ns non-significant

Previous studies suggested that sLDL-R is generated mainly due to shedding from cell surfaces by peptidases MMP-14, ADAM-17, and BMP-1 [22–25]. In addition, alternatively spliced variants of LDL-R have been reported although it is unknown whether those variants generate sLDL-R. Therefore, to explore the mechanism behind TNF-α induced sLDL-R in HepG2, we analyzed the gene expression of LDL-R as well as LDL-R cleaving peptidases MMP-14, ADAM-17, and BMP-1 [22–25]. For qPCR analyses LDL-R variants, we used 3 primers targeting exons coding for amino-terminus (exon 1–2), exons coding for the membrane-spanning domain (exons 15–16), and exons coding for carboxyl-terminus (exon 17–18) of the LDL-R gene. As shown in Fig. 3A–C, treatment of HepG2 cells with TNF-α significantly upregulated the gene expression of LDL-R, and the induction was similar in all three target regions suggesting that an alternatively spliced variant leading to sLDL-R is negligible. Furthermore, we showed that the gene expression of peptidases MMP-14 and ADAM-17 was upregulated, while gene expression of BMP-1 was unaltered, in HepG2 cells following TNF-α stimulation (Fig. 3D–F). Moreover, the level of sLDL-R induced by TNF-α was reduced when HepG2 cells were co-stimulated with TMI-1 (an inhibitor of ADAM-17 and MMP-14) but not with BMP-1 inhibitor (UK383367) (Fig. 3G). These findings suggest that the TNF-α induced generation of sLDL-R from HepG2 cells is resulted from membrane shedding by increased activity of ADAM-17 and MMP-14.

### Soluble LDL-R (sLDL-R) inhibits LDL uptake by HepG2 cells

**Fig. 4:**
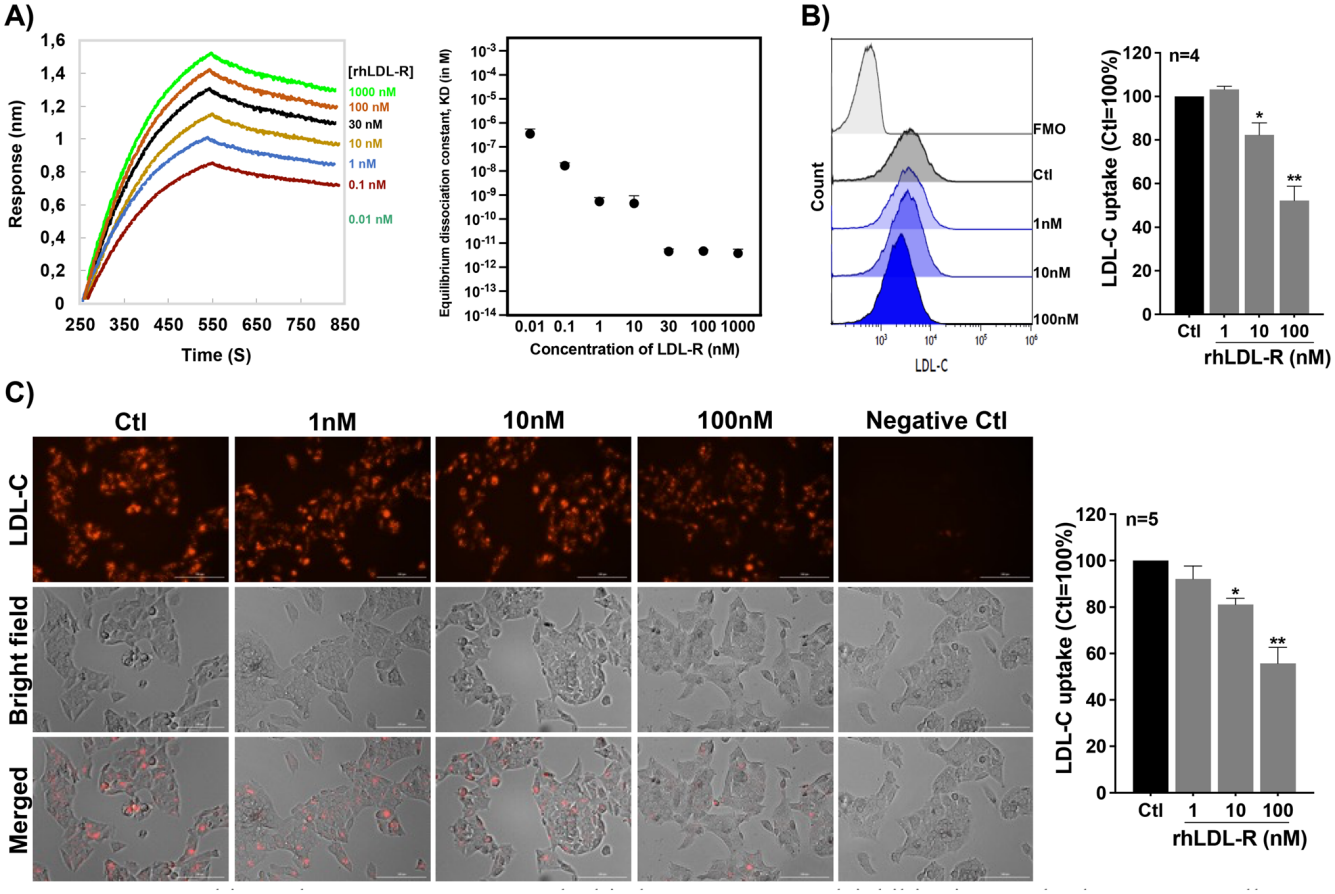
Recombinant human LDL-R strongly binds to LDL-C and inhibits its uptake by HepG2 cells. **A** Representative Sensogram and a plot of KD values from 3 independent analyses. **B** A representative histogram and a bar graph depicting the LDL-C uptake by HepG2 cells in the presence of rhLDL-R. **C** Representative microscopic images and quantification of LDL-C uptake by HepG2 cells in the presence of rhLDL-R. *p < 0.05, **p < 0.01. AU arbitrary unit, FMO fluorescence minus one, ns non-significant

The ectodomain shedding of LDL-R by ADAM-17 and MMP-14 results in a sLDL-R, with molecular size between 120 and 140 kDa, which contains the LDL-C binding region of LDL-R [23–25]. Furthermore, gel filtration chromatographic analyses of human plasma revealed that high quantity of sLDL-R was found in LDL-C fraction. In this study, we aimed to investigate the LDL-C binding characteristics of sLDL-R and its biological relevance. To do this, we used a recombinant human LDL-R (rhLDL-R) protein that is composed of the extracellular portion of the receptor and has a molecular weight of 120–145 kDa. First, we measured the binding kinetics of the rhLDL-R to LDL-C using BLI-based affinity assay, and our results indicated a dose-dependent increase in affinity of LDL-C binding to rhLDL-R. As depicted in Fig. 4A, we observed that KD (inversely proportional to affinity) values were about 1 μM–1 pM at different concentrations and the affinity of LDL-C to rhLDL-R remained high at the higher concentrations. Next, we incubated HepG2 cells with pHrodo Red labeled LDL-C in the presence or absence of rhLDL-R in the medium. We found that the rhLDL-R significantly reduced LDL-C uptake by HepG2 cells in a dose-dependent manner (Fig. 4B, C). The rhLDL-R also interfered with LDL-C uptake by human vascular endothelial cells (Supplementary Fig. 2). These findings suggest that soluble extracellular LDL-R can stably bind LDL-C and consequently interfere with LDL-C uptake by HepG2 cells.

### Circulating LDL-R, future myocardial infarction (MI), and correlations with plasma cholesterol in humans

To further investigate the interplay between TNF-α, sLDL-R, and LDL-C clearance in pathophysiologic context, we conducted a nested case-control study using the VIP and MONICA cohorts. We included 584 study participants (292 MI cases and 292 age- and sex-matched controls) and analyzed baseline plasma levels of sLDL-R and TNF-α. The socio-demographic and clinical characteristics of the study participants have been described previously [26]. Briefly, two-thirds of the study participants were men (76.4%), and the median age was about 60 years. While distribution of age and sex was proportional between cases and controls by design, the prevalence of obesity, high blood pressure, diabetes mellitus, current smoking, and high blood cholesterol was higher in cases compared to controls (Table 1).

**Table 1:** Clinical characteristics of study participants at baseline

	**Cases (*n* = 292)**	**Controls (*n* = 292)**
Age^a^	59.7 (50.1–60.1)	59.8 (50.1–60.1)
Sex (male)	76.4%	76.4%
Physical inactivity	77.1%	75.9%
Obesity	18.8%	12.0%
Hypertension	40.6%	25.2%
Diabetes Mellitus	11.8%	2.4%
Current smoking	34.2%	22.6%
Total cholesterol^a,b^	6.31 (5.53–7.04)	5.81 (5.13–6.45)

^a^Shown as median and inter-quartile range (IQR)

^b^Reported in mmol/L

**Fig. 5:**
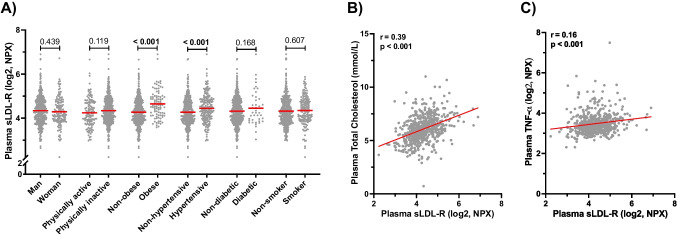
Plasma sLDL-R level and its relationship with clinical characteristics of study participants at baseline. **A** A scatter plot showing the distribution/comparison of plasma sLDL-R in categories of clinical characteristics of study participants at baseline. Scatter plots showing the correlation between plasma sLDL-R concentration and total cholesterol (**B**) TNF-α (**C**). NPX normalized protein expression

For this study, the concentration of LDL-R and TNF-α in plasma was analyzed using Olink proteomics (Uppsala, Sweden), and the data was reported on log2 scale. The sLDL-R concentration was normally distributed in the current study population with mean and SD values of 4.33 and 0.62, respectively (Supplementary Fig. 3). As presented in Fig. 5A, the mean plasma concentration of LDL-R was significantly higher in obese participants compared to non-obese participants (*p*-value < 0.001) at baseline. In addition, baseline plasma sLDL-R was significantly higher in hypertensive participants compared to non-hypertensive participants (*p*-value < 0.001). We found no statistically significant differences in mean plasma levels of LDL-R when comparing the categories of sex, physical inactivity, diabetes status, and current smocking status (Fig. 5A). We also found no significant correlation between age and plasma sLDL-R levels (*r* = 0.03, *p* = 0.531). However, there was a significant positive correlation between plasma total cholesterol and LDL-R levels (*r* = 0.39, *p*-value < 0.001) at baseline (Fig. 5B). Moreover, we found a statistically significant positive correlation between plasma sLDL-R and pro-inflammatory cytokine TNF-α (*r* = 0.16, *p*-value < 0.001) (Fig. 5C).

**Fig. 6:**
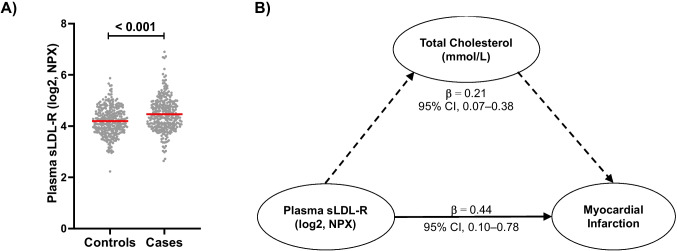
Plasma sLDL-R level is associated with increased risk of future MI, and its association is mediated by increase in plasma cholesterol level. **A** Scatter plot showing the distribution of plasma sLDL-R concentration in cases and controls (*n* = 584). **B** Schematic illustration of the direct and indirect effects of plasma sLDL-R on risk of future MI (*n* = 543)

We found that the plasma level of LDL-R was significantly higher in MI patients than their matched controls (Fig. 6A). Moreover, logistic regression analyses showed that a two-fold increase in plasma sLDL-R concentration is associated with 2.1-times higher risk of MI (95% CI = 1.6–2.8). Given the potential of sLDL-R in interfering with LDL-C clearance and hence contributing to elevated circulating cholesterol, we hypothesized that part of the association between plasma sLDL-R and MI could be due to increase in plasma cholesterol. To investigate this, we first performed a multi-variable regression analysis between plasma sLDL-R and MI by including other variables such as age, sex, obesity, physical inactivity, hypertension, DM status, and current smoking status. As such, we determined that the AOR was 1.9 (95% CI = 1.4–2.6). In the next model, we included total cholesterol and found that the AOR was 1.55 (95% CI = 1.10–2.18). The reduction in OR following adjustment to total cholesterol indicates that the effect of sLDL-R on MI is partly carried through total cholesterol. To evaluate this relationship further statistically, we performed mediation analyses using the approach by Hayes and Preacher [20] that can enable us to estimate the direct and indirect effects of sLDL-R on MI. As shown in Fig. 6B, the direct effect of elevated plasma sLDL-R on risk of future MI was 0.44 (95% CI, 0.10–0.78), whereas its indirect effect was 0.21 (95% CI, 0.07–0.38). Based on these values, we estimated that the proportion mediated by plasma total cholesterol in the association of plasma sLDL-R to MI was 0.39.

## Discussion

This study reports that TNF-α induces release of sLDL-R and such form of the receptor can bind to the LDL-C inhibiting its clearance by human hepatoma cells in vitro. In human plasma, concentration of sLDL-R was found to be positively correlated with circulating total cholesterol level. Furthermore, plasma level of sLDL-R was strongly associated with the risk of future MI and that a significant proportion of the association can be explained by elevation in total cholesterol.

We demonstrated that the pro-inflammatory cytokine TNF-α induces release of sLDL-R in HepG2 cells in a dose-dependent manner. We found that pharmacologic inhibition of previously known LDL-R cleaving enzymes ADAM-17 and MMP-14 prevents TNF-α induced release of sLDL-R from HepG2 cells. Interestingly, when analyzed at 48 h, the level of LDL-R on surface of HepG2 cells was not affected in response to TNF-α which was further supported by functional assays that LDL-C uptake was comparable between TNF-α treated and untreated cells. It turns out that TNF-α also induces a rapid (30 min after stimulation) and persistent upregulation of LDL-R gene expression in HepG2 cells, which is consistent with previous findings [27]. Our findings suggest that LDL-R shedding induced by TNF-α is counteracted by transcriptional upregulation that results in normalization of the level of functional LDL-R on cell surfaces. In vitro experiments using PMA (Phorbol 12-myristate 13-acetate), the commonly used inducer of LDL-R shedding, showed marked decline in the level of LDL-R expressed on hepatocytes only during the first 1–2 h of treatment which is then reversed in later time points suggesting for compensation from upregulation in gene transcription [25]. It is well known that LDL-R gene transcription is principally regulated by SREBP2 whose activity is tightly regulated by intracellular cholesterol availability which in turn is dependent of either de novo biosynthesis or endocytosis of cholesterol [10, 11]. However, engagement of TNF-receptor on hepatocytes can lead to activation of downstream signaling pathways involving SREBP2 [28]. Recent study using vascular endothelial cells demonstrated that TNF-α decreases intracellular cholesterol pool leading to enhanced SREBP2 activation [29]. A cholesterol-independent SREBP2 regulation by TNF-α has also been shown in macrophages [30]. That TNF-α also generates sLDL-R shedding from other cell types such as ECs, human monocytes, and macrophages indicate its potential to act systemically in vivo and result in accumulation of sLDL-R in the circulation.

Metalloproteases including ADAM-17 and MMP-14 cleave the LDL-R by the surface of cells releasing a 120–140 kDa soluble form that contains the LDL-C binding region of LDL-R [23–25]. Additionally, a soluble version with a considerably lower molecular weight (35–40 kDa) but containing the LDL-C binding region has been reported [22, 31]. These could potentially result in sLDL-R with intact LDL-C binding capability in vivo. Indeed, a gel filtration chromatography using human plasma detected high levels of sLDL-R in LDL-C fractions as well as in the fractions of VLDL and LDL remnants [23]. In agreement to this observation, we demonstrated that a soluble form of the extracellular region of LDL-R can bind to LDL-C with strong affinities, and that the complex formed has marked stability. Moreover, we could show that the presence of a soluble form of LDL-R inhibited endocytosis of LDL-C by HepG2 cells in a dose-dependent manner. It has been speculated previously that hypercholesterolemia associated with increased sLDL-R in plasma could indicate diminished level of LDL-R on surface of hepatocytes due to shedding rendering the cells incapable of removing LDL-C from the circulation [14]. However, there is paucity of evidence regarding the level of cell surface LDL-R expression in vivo during high sLDL-R release, and the impact of the sLDL-R released has not been considered. Here, we propose an alternative explanation that accumulation of sLDL-R in circulation may hinder clearance of LDL-C by hepatocytes, which otherwise possess normal level of surface LDL-R, due to competition with the membrane-bound receptor. Moreover, given the stability of the complex formed by sLDL-R and LDL-C as indicated by the very low off-rates, it is likely that this could lead to increased half-life of LDL-C in the circulation and lead to hypercholesterolemia and associated pathologies such as atherosclerotic cardiovascular diseases.

Our nested case-control study revealed that sLDL-R concentration in plasma is strongly correlated with circulating total cholesterol level at baseline. This is consistent to previous reports [14–16]. A moderate, but significant, positive correlation was also evident between sLDL-R and TNF-α levels at baseline. It turned out that baseline sLDL-R concentration in plasma is strongly associated with increased risk of future MI. This association remained statistically significant in multivariate logistic regression analyses suggesting that baseline sLDL-R is an independent predictor of future MI. This, to the best of our knowledge, is the first study to report an association between sLDL-R and incidence of MI. Interestingly, we noticed that the risk of future MI associated with a twofold increase in sLDL-R was markedly reduced when adjusted for baseline total cholesterol level (from 90 to 55%) suggesting for a possible mediated effect. Because LDL-R/cholesterol pathway is causally linked to MI [1], and that increase in plasma sLDL-R is associated with increase in total cholesterol, we intended to clarify the association between sLDL-R and future MI by performing a mediation analysis using the approach by Hayes and Preacher [20]. Through our analyses, we found that a significant proportion (nearly 40%) of the association between sLDL-R and incidence of future MI is carried through elevation in plasma total cholesterol levels. Therefore, targeting the release of sLDL-R or its binding to LDL-C in the circulation could theoretically be exploited therapeutically in the management of hypercholesterolemia and MI. In this regard, silencing expression of MMP14 in atherosclerotic mice models have shown to significantly reduce plasma sLDL-R level and cholesteryl ester accumulation in aorta of atherosclerotic mice models [23]. The significance of enhanced hepatic LDL-R expression in lipid lowering has been recognized before. For instance, part of the lipid lowering effects of statins has been attributed to increased hepatic LDL-R level either by direct transcriptional induction or through inhibition of negative regulators of LDL-R such as IDOL [3]. In addition, the FOURIER trial has recently demonstrated that blocking PCSK9, a negative regulator of LDL-R, results in 15% reduction of major adverse cardiovascular events [32]. Hence, therapeutic targeting of LDL-R shedding could provide additional avenue for strategies of lowering lipids and associated pathophysiology.

In conclusion, our in vitro experiments revealed mechanisms through which the pro-inflammatory mediator TNF-α induces sLDL-R release from HepG2 cells. In addition, we demonstrated the inhibitory effect of a soluble form of LDL-R on the hepatocytic uptake of LDL-C. Furthermore, using a nested case-control study, we provide evidence that plasma level of sLDL-R at baseline was strongly associated with future myocardial infarction (MI) and that a significant proportion of the association can be explained by elevation in plasma cholesterol level.

### Supplementary Information

Below is the link to the electronic supplementary material.

Supplementary file1 (DOCX 12.7 KB)

## Data Availability

The data used and/or analyzed in this study are contained within the manuscript.
