# Supplementary material for: Soluble LDL-receptor is induced by TNF-α and inhibits hepatocytic clearance of LDL-cholesterol
Source: J Mol Med (Berl). 2023 Oct 20;101(12):1615–26. doi: 10.1007/s00109-023-02379-4 (PMC10697900; doi:10.1007/s00109-023-02379-4)
Supplement: Supplementary file 1 — Supplementary file1 (DOCX 12.7 KB) [file 109_2023_2379_MOESM1_ESM.docx]

**Supplementary figures**

**Supplementary Figure 1.** Treatment with TNF-α (50ng/ml) induces release of sLDL-R to the culture

supernatants of **A)** HUVECs, **B)** THP-1 cells and **C)** PMA-differentiated THP-1 cells. THP-1 cells were

differentiated using PMA (10nM for 24h) prior to treatment with TNF-α. ****p<0.0001. PMA=Phorbol-12-

myristate-13-acetate

**Supplementary Figure 2.** Recombinant human LDL-R inhibits LDL-c uptake by human vascular

endothelial cells. The bar graph depicting LDL-C uptake by HUVECs in the presence of rhLDL-R.

***p<0.001.

**Supplementary Figure 3.** A histogram showing the frequency distribution and normality statistics of

plasma sLDL-R in all the study participants.
